# Repair of a Grynfeltt-Lesshaft hernia with the PROCEED™ VENTRAL PATCH: a case report

**DOI:** 10.1186/s40792-018-0456-x

**Published:** 2018-05-30

**Authors:** Torben Glatz, Hannes Neeff, Philipp Holzner, Stefan Fichtner-Feigl, Oliver Thomusch

**Affiliations:** Department of General and Visceral Surgery, University of Freiburg, Hugstetter Str. 55, 79106 Freiburg im Breisgau, Germany

**Keywords:** Grynfeltt-Lesshaft hernia, Lumbar hernia, PROCEED™ VENTRAL PATCH

## Abstract

**Background:**

Primary hernias in the triangle of Grynfeltt are very rare and therefore pose a difficulty in diagnosis and treatment. Due to the lack of systematic studies, the surgical approach must be chosen individually for each patient. Here, we describe an easy and safe surgical approach.

**Case presentation:**

We report the case of a 53-year-old male patient with a history of mental disability and pronounced scoliosis, who presented with a Grynfeltt-Lesshaft hernia with protrusion of the ascending colon and the right ureter.

The hernia was repaired via a dorsal, extraperitoneal approach. The hernia gap with a diameter of approximately 1 cm was closed with insertion of a 6.4 × 6.4 cm PROCEED™ VENTRAL PATCH (Ethicon, Norderstedt, Germany). The operating time was 33 min and the patient was discharged the next day and showed no signs of recurrence at 1-year follow up.

**Conclusion:**

The technique described here is favorable because it requires very little dissection of the surrounding tissue and no trans-/intraabdominal dissection. The technique was chosen in this particular case to guarantee a fast postoperative recovery and prompt hospital discharge.

## Background

Lumbar hernias are a very rare cause of abdominal complaints, and there is no standardized surgical strategy. Here, we present the case of a mentally disabled patient with a primary Grynfeltt-Lesshaft hernia with herniation of the ascending colon causing constipation and abdominal pain. Due to the mental disability and the severe symptoms of the hernia, a safe surgical approach with fast postoperative recovery was required.

## Case presentation

**Fig. 1:**
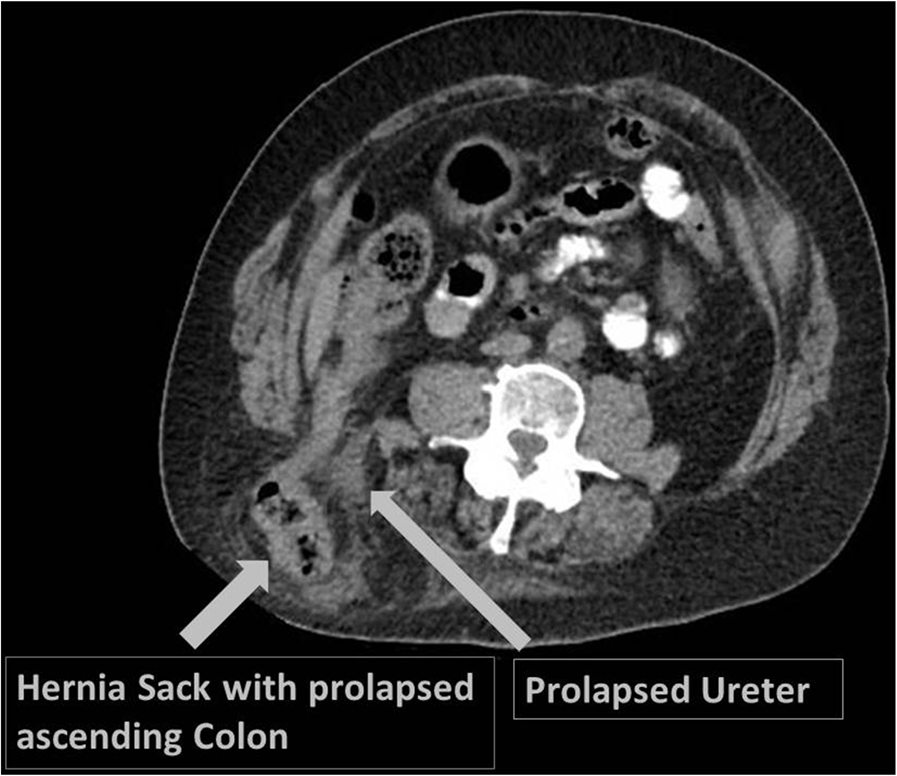
Preoperative CT revealed a large Grynfeltt hernia with prolapse of the ascending colon and the right ureter with consecutive dilatation of the renal pelvis

The 53-year-old patient with a history of mental disability and pronounced scoliosis presented repeatedly to our emergency department with intermittent episodes of constipation and abdominal pain. Physical examination revealed a double-sided inguinal hernia without signs of incarceration and a right lumbar protrusion with the clinical appearance of a soft tissue mass. After several episodes of abdominal complaints, a computed tomography of the abdomen was conducted, which revealed a large herniation in the upper lumbar triangle with protrusion of the ascending colon and the right ureter with consecutive dilatation of the proximal ureter and the renal pelvis (Fig. 1). The lumbar hernia was reduced at the bedside, and the patient was admitted for early surgical hernia repair.

**Fig. 2:**
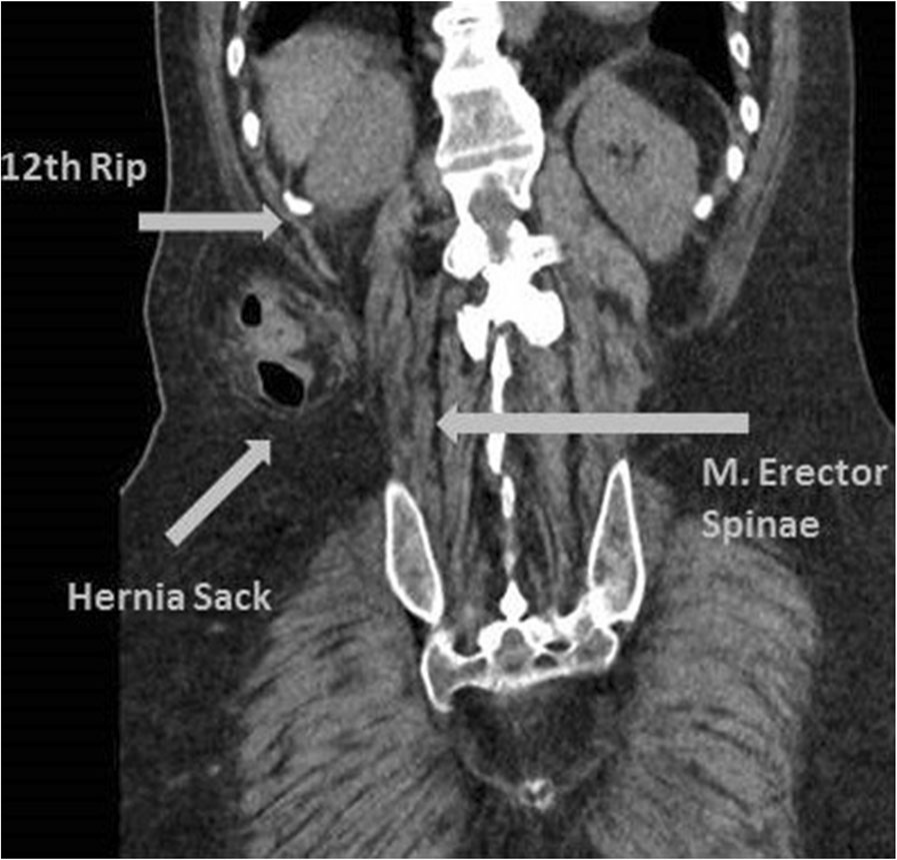
The hernia sack prolapsed in the superior lumbar triangle between the M. Erector Spinae, the internal oblique and the 12th rip

**Fig. 3:**
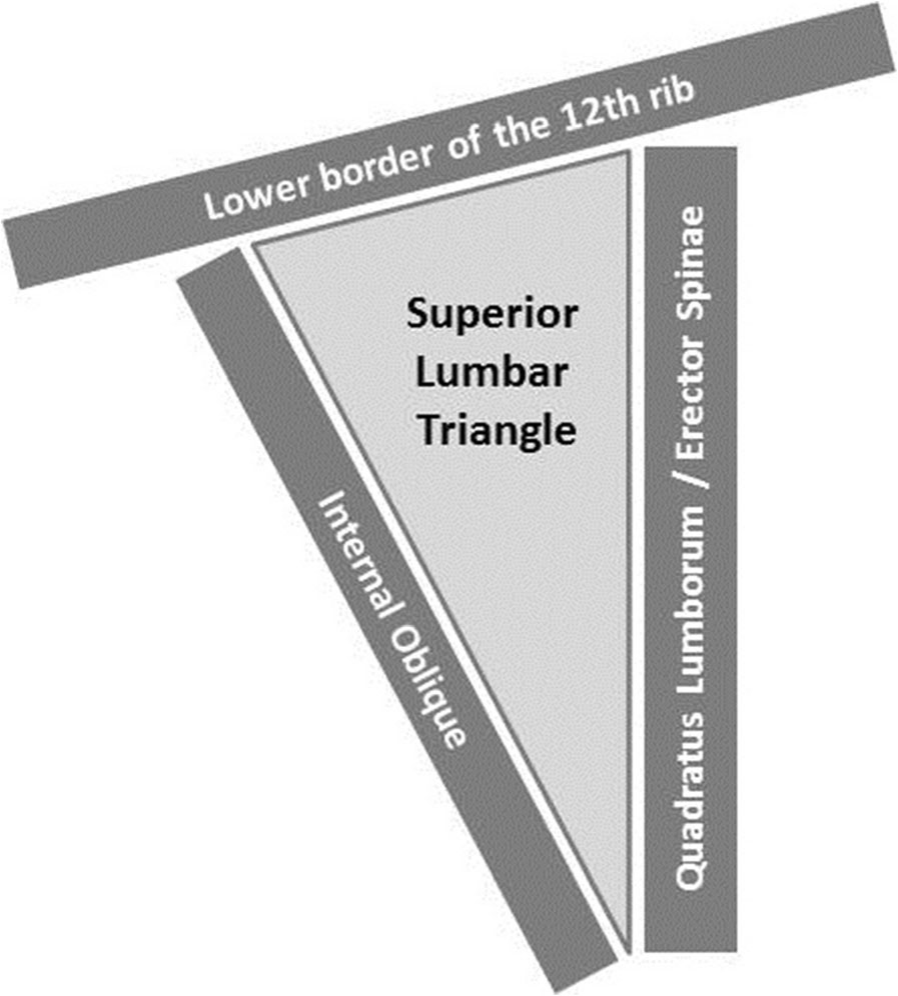
Schematic display of the superior lumbar triangle. The triangle is inverted and partially covered by the latissimus dorsi. The base is formed by the lower border of the 12th rib the triangle is anteriorly bounded by the internal oblique muscle and posteriorly by the quadratus lumborum and erector spinae muscles

**Fig. 4:**
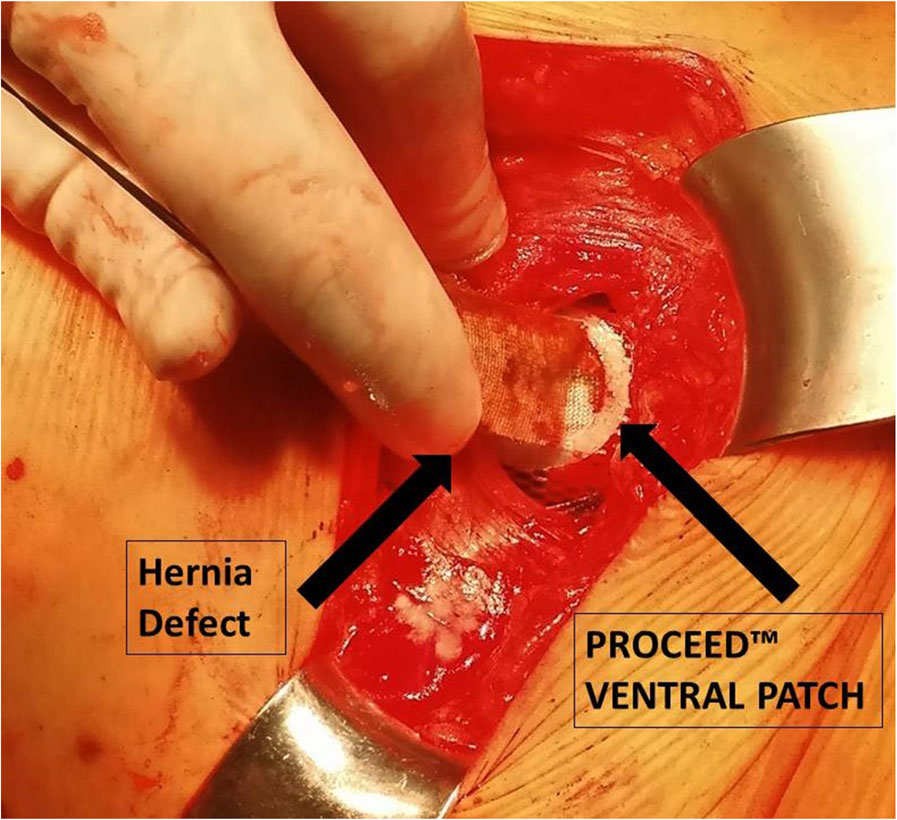
Surgical repair of the hernia was performed with the patient in a lateral position via a dorsal approach and insertion of a 6.4 × 6.4 cm PROCEED™ VENTRAL PATCH (Ethicon, Norderstedt, Germany)

After routine medical check-up and informed consent of the legal guardian, the patient was operated under general anesthesia. The patient was placed in a left lateral position. A dorsal transverse incision of 6 cm between the 12th rib and iliac crest was made. The superior lumbar triangle with the bordering structures was visualized. The triangle is inverted with the top pointing towards the iliac crest and is partially covered by the latissimus dorsi. The base is formed by the lower border of the 12th rib and the posterior inferior serratus muscle (cranial); the triangle is anteriorly bounded by the internal oblique muscle and posteriorly by the quadratus lumborum and erector spinae muscles (the anatomy of the superior lumbar triangle is described in Figs. 2 and 3). The transversalis fascia forms the floor, which was weakened in our patient and contained the hernia gap with a diameter of approximately 1 cm. The hernia sack was dissected and reduced. To do so without risking any injury of the herniating colon, the internal oblique muscle was notched. The retroperitoneal fat was easily separated from the lower surface of the transversalis fascia by digital adhesiolysis. The gap was closed with insertion of a 6.4 × 6.4 cm PROCEED™ VENTRAL PATCH (Ethicon, Norderstedt, Germany) in sublay technique with an overlap of 3 cm to all sides. The lower part of the patch was laid out flat and tension free between the transversalis fascia and the retroperitoneal fat (Fig. 4). The straps were pulled to ensure the correct position of the patch and were then sutured to the fascia with non-absorbable sutures, after which the overlaps were resected. The muscles and fascia were adapted with absorbable sutures and the wound was closed. Drain placement was not necessary. The operating time was 33 min, and the patient was discharged the next day and showed no signs of recurrence at 1-year follow up.

## Discussion and conclusion

A review of the literature reveals only a handful of case reports, describing various open and laparoscopic techniques for the closure of lumbar hernias. Due to the rare appearance, there is hardly any evidence supporting one or the other technique [1].

Considering the general condition of our patient and the need for an urgent operation, we decided against laparoscopy, but instead for an open, extraperitoneal, dorsal approach with insertion of a self-expanding, partially absorbable, flexible laminate mesh device, very similar to the approach described by Solaini et al. [2].

The effective use of the PROCEED™ VENTRAL PATCH has been demonstrated for umbilical hernias [3] and likewise small incisional hernias. We could demonstrate that the patch can be also effectively used for closure of hernias in the Grynfellt-Triangle via a dorsal approach.

Up to now, there is no retrospective study or randomized controlled trial analyzing the results of the different surgical techniques for closure of a primary lumbar hernia, and due to the rare appearance, there will probably never be one. Therefore, the surgical approach has been chosen individually for each patient with consideration of the particular situation.

In 2013, Suarez et al. recommended a laparoscopic approach to hernias in the triangle of Grynfellt with the main argument of faster postoperative recovery and less postoperative pain and consumption of pain medication [4]. This recommendation is based mostly on the result of a randomized controlled trial comparing open and laparoscopic repair of secondary lumbar hernias, which reveals beneficial results of the laparoscopic approach [5]. However, this study was carried out with patients suffering from incisional hernias and is therefore not comparable with our case of a primary Grynfeltt-Lesshaft hernia. The trial reports a mean operating time of 71 min, a postoperative morbidity of 86%, and a mean hospital stay of 7 days for the open approach. These figures certainly do not compare to the technique described here.

Our approach is favorable; hence, it requires almost no dissection of the surrounding tissue and muscles and no (laparoscopic) intraabdominal dissection. It can also be performed by any surgeon familiar with more common hernias (e.g., umbilical) even with limited experience with lumbar hernias in a very short time and therefore is very safe. Due to the position and size of the incision and the limited need for dissection, postoperative recovery is fast and incisional pain is acceptable. The overlap of the mesh was about 3 cm to all sides. Due to the small gap and the strong tissue around the hernia, we refrained from further preparation to insert a larger mesh with a wider overlap. We were able to discharge the patient who had a mental disability the next day. A mentally and physically healthy patient could have been presumably treated in an outpatient setting. Our operative technique is certainly a feasible alternative to the laparoscopic approach. In this particular case, it was our chosen approach due to the need for an urgent operation and to guarantee a fast postoperative recovery and prompt hospital discharge.
